# Multi-channel masked autoencoder and comprehensive evaluations for reconstructing 12-lead ECG from arbitrary single-lead ECG

**DOI:** 10.1038/s44325-024-00036-4

**Published:** 2024-12-04

**Authors:** Jiarong Chen, Wanqing Wu, Tong Liu, Shenda Hong

**Affiliations:** 1 National Institute of Health Data Science, Peking University, Beijing, China; 2 School of Biomedical Engineering, Sun Yat-sen University, Shenzhen, Guangdong China; 3 Department of Micro/Nano Electronics and MoE Key Lab of Artificial Intelligence, Shanghai Jiao Tong University, Shanghai, China; 4 Tianjin Key Laboratory of lonic-Molecular Function of Cardiovascular Disease, Department of Cardiology, Tianjin Institute of Cardiology, Second Hospital of Tianjin Medical University, Tianjin, China; 5 Institute of Medical Technology, Health Science Center of Peking University, Beijing, China; 6 Institute for Artificial Intelligence, Peking University, Beijing, China

**Keywords:** Arrhythmias, Arrhythmias

## Abstract

Electrocardiogram (ECG) has emerged as a widely accepted diagnostic instrument for cardiovascular diseases (CVD). The standard clinical 12-lead ECG configuration causes considerable inconvenience and discomfort, while wearable devices offers a more practical alternative. To reduce information gap between 12-lead ECG and single-lead ECG, this study proposes a multi-channel masked autoencoder (MCMA) for reconstructing 12-Lead ECG from arbitrary single-lead ECG, and a comprehensive evaluation benchmark, ECGGenEval, encompass the signal-level, feature-level, and diagnostic-level evaluations. MCMA can achieve the state-of-the-art performance. In the signal-level evaluation, the mean square errors of 0.0175 and 0.0654, Pearson correlation coefficients of 0.7772 and 0.7287. In the feature-level evaluation, the average standard deviation of the mean heart rate across the generated 12-lead ECG is 1.0481, the coefficient of variation is 1.58%, and the range is 3.2874. In the diagnostic-level evaluation, the average F1-score with two generated 12-lead ECG from different single-lead ECG are 0.8233 and 0.8410.

## Introduction

The cardiovascular disease (CVD)^1,2^ contributes the leading mortality all around the world. Moreover, the prevalence rate continues to show an upward trend in the developing areas in the past decades^3^, posing a great challenge for researchers and cardiologists to address. In clinical practice, clinicians need to adopt some characterization tools^4^ to diagnose cardiovascular disease, and one of the most popular tools is the standard 12-lead electrocardiogram (ECG). The significant advancements in deep learning have enabled certain researchers to develop models capable of achieving cardiologist-level proficiency in interpreting 12-lead electrocardiograms (ECGs). For instance, Ribeiro et al. have successfully trained such a cardiologist-like model, as detailed in their study^5^. In conclusion, the 12-lead ECG can provide comprehensive cardiac information from various views for doctors and classification models, playing an essential role in cardiac healthcare.

However, the 12-lead ECG signal collection process puts at least 10 electrodes on the user’s surface, which causes considerable inconvenience and discomfort for users, and make long-term cardiac health monitoring difficult. Up to now, the standard 12-lead ECG is traditionally used in the hospital for short-term diagnosis, usually lasting about 1 min, while long-term monitoring^6^ is essential for capturing the paroxysmal cardiac abnormalities. Consequently, the pursuit of user-friendly devices capable of capturing ubiquitous electrocardiogram (ECG) signals is a priority for both researchers and markets, including patch^6–8^, smartwatch^9–11^, and armband^12–14^. Further, the single-lead ECG has been used for cardiac abnormality classification, such as the lead I ECG for the Atrial Fibrillation^15^, the lead V1 ECG for the Brugada Syndrome^16^, and the lead aVR ECG for the Sinus Bradycardia^17^. While wearable devices offer the advantage of ambulatory monitoring by collecting single-lead ECG signals, they do not match the diagnostic depth of a standard 12-lead ECG. The limitation arises from these devices capture the heart’s electrical activity from a restricted subset of perspectives, which may not provide a comprehensive assessment of cardiac health.

It is of great importance to strike a harmonious balance between clinical effectiveness and application feasibility. On the one hand, the clinical standard 12-lead ECG can comprehensively measure cardiac health^5^, but it causes somewhat inconvenience and discomfort. On the other hand, wearable devices have been a popular choice for users, but they are with limited clinical importance. Then, many researchers tried to reduce the gap between the reduced-lead and 12-lead ECG, like the challenge proposed by Reyna et al.^18^. The challenge asks to access the diagnostic potential of the reduced-lead ECG, including 6-lead, 4-lead, 3-lead, and 2-lead ECG. In this challenge, Nejedly et al.^19^ adopt the ensemble learning, residual network, and attention mechanism to achieve state-of-the-art performance, and similarly in these researches^20–23^. Unfortunately, these mentioned studies only focus on the classification performance, merely providing an indirect approach to reduce the gap between the reduced-lead and 12-lead ECG.

Subsequently, some researchers try to provide a direct approach to reduce the gap between the reduce-lead (Specifically, single-lead) and 12-lead ECG, that is, reconstructing 12-lead ECG with the reduced-lead ECG^24–33^. Prior works managed to explore transformation between the Frank lead and the standard 12-lead ECG, in which the inverse Dower matrix is released by Edenbrandt et al.^24^, and it turns 12-lead ECG into 3-dimensional Vectorcardiogram. Nelwan et al.^25^ attempt to reconstruct 12-lead ECG from reduced lead sets. The experimental findings indicate a strong correlation coefficient of ~0.932 when one or two precordial leads are excluded from the lead set. Maheshwari et al.^26^ adopt a solution for reconstructing 12-lead ECG from 3-lead ECG, and the reconstruction score is about 0.9187 in the testing phase. However, the assumption of dominantly linear relationship between ECG vectors can not fit the human heart electrical conduction system. Some researchers adopted autoencoders with different model architectures, such as Atoui et al.^27^ proposed Artificial Neural Network (ANN), and successfully realized the generation process of 3-lead ECG to the remaining 5 chest leads. This work and the following work all adopt the training idea of automatic encoders, including Sohn et al.^28^ used LSTM; Gundlapalle et al.^29^ combined CNN and LSTM; Garg et al.^30^ combined the attention mechanism in autoencoder, thereby improving the feature expression ability. Generative adversarial network (GAN)^34^ also attracts a number of research attention, such as refs. ^31–^^33^. Lee et al.^31^ adopt the conditional generative adversarial network(CGAN) to explore the feasibility of converting limb leads into chest leads. It is worth mentioned that the input of CGAN is ECG, instead of the random noise in the traditional GAN. The average structural similarity index (SSIM) between the generated ECG signal and the real ECG signal is 0.92, and the percent root mean square difference (PRD) is only 7.21%. Seo et al.^32^ also use the CGAN for reconstructing 12-lead, and the Mean Absolute Error (MAE) between the generated and real ECG signals is only 0.25. Joo et al.^33^ proposes a novel CGAN that consists of two generators, and achieves good reconstruction performance, like the root mean square error between the generated and real 12-lead ECG is 0.32. Additionally, our previous work^35^ also uses this method to reconstruct 12-lead ECG from lead I ECG. However, the training instability and poor diversity make generating adversarial networks to difficultly address this reconstruction task, and most of the above-mentioned studies are limited flexible, since they only work on a fixed limb lead^30,32,35^. Chen et al.^36^ propose a novel framework to establish Electrocardio panorama; however, only the 12-lead ECG signals are considered useful, while the remaining non-standard lead signals are deemed meaningless. Consequently, there is a critical need to investigate methods for reconstructing the 12-lead ECG from an arbitrary single-lead ECG, While these methodologies are capable of approximating the reconstruction of a 12-lead electrocardiogram (ECG) from limited-lead inputs, there remains a significant research gap that needs to be addressed in the domain of 12-lead ECG reconstruction. Firstly, the traditional generative models usually focus on the fixed single-lead, instead of arbitrary single-lead ECG. Secondly, the related works^27–33,35^ lack a comprehensive evaluation benchmark, mainly focus on the signal-level evaluation. Therefore, the contributions in this study are as follows:

- This study proposes a multi-channel masked autoencoer, MCMA, and it can convert arbitrary single-lead ECG into 12-lead ECG.
- This study designs a comprehensive evaluation benchmark, ECGGenEval, including signal-level, feature-level, and diagnostic-level evaluation.
- MCMA can achieve state-of-the-art reconstruction performance in the ECGGenEval across the internal and external testing datasets, with a mean square error of 0.0175 and a Pearson correlation coefficient of 0.7772 in the internal testing dataset.

In a word, MCMA demonstrates its efficacy in reconstructing a 12-lead ECG from a single lead, thereby offering significant potential to augment the capabilities of wearable health monitoring devices in the digital health era. This advancement is poised to improve the diagnostic and monitoring capabilities of these devices, ensuring more accurate and accessible health assessments for users.

## Method

### ECG background

ECG capture the electrical activity of the heart, characterized by distinct waveforms such as the P-wave, QRS-complex, and T-wave. The standard 12-lead ECG has been a prevalent diagnostic tool in clinical practice due to its ability to provide a comprehensive view of cardiac function. This tool, however, requires the placement of 10 electrodes on the body’s surface. The electrode positioning in the 12-lead ECG is detailed in Table 1.

**Table 1:** ECG background: the standard electrode configuration in the standard 12-lead ECG

Lead	Electrode Position
I	Left Arm, Right Arm
II	Left Foot, Right Arm
III	Left Foot, Left Arm
aVR	Right Arm
aVL	Left Arm
aVF	Left Foot
V1	4th intercostal space at the right sternal border
V2	4th intercostal space at the left sternal border
V3	Midpoint between V2 and V4
V4	5th intercostal space at the midclavicular line
V5	Lateral to V4, at the left midaxillary line
V6	Lateral to V5, at the left midaxillary line

### Dataset

This study conducts a large-scale 12-lead ECG datasets, consisting of 28,833 recordings from three public 12-lead ECG datasets, i.e., PTB-XL^37,38^, CPSC2018^39^, and CODE-test^5^. The proposed framework is trained and validated with PTB-XL initially, and using the internal and two external testing datasets to further prove its feasibility.

PTB-XL^37,38^ is used for model training, validating, and testing. As a large dataset, PTB-XL involves 21,799 clinical 10-s 12-lead ECG signals, and the sampling frequency is 500 Hz. Based on the clinical standard, this dataset includes 71 kinds of ECG statements. As recommended, this study adopts the standard tenfold setting, in which the folds from the 1st fold to the 8th fold is the training set, and the 9th fold and the 10th fold act as the validation set and testing set, respectively. The ratio for training:validation: and testing is about 8:1:1.

CPSC2018^39^ is used as an external testing set since the data distribution and information do not appear in model training and choosing. CPSC2018 contains 6877 12-lead ECG, and these lengths varied from 6 s to 60 s with 500 Hz in sampling frequency.

CODE-test is also used as an external testing set, particularly for diagnostic-level evaluation. CODE-test includes 827 12-lead ECG collected from different patients with different arrhythmia. Ribeiro et al.^5^ contributed a trained cardiologist-level classification model for this testing dataset.

Table 2 presents the data distribution for the signal-level and feature-level evaluation in PTB-XL and CPSC2018. Table 3 presents the data distribution for the diagnostic-level evaluation in CODE-test, including 6 distinguished arrhythmia types in this dataset.

**Table 2:** The data distribution of PTB-XL and CPSC2018, and these datasets are used for signal-level and feature-level evaluation

Dataset	Role	Number
PTB-XL	Training Set	87,200
	Validation Set	10,965
CPSC2018	Internal testing set	11,015
	External testing set	55,999

**Table 3:** The data distribution of CODE-test, and it is used for the diagnostic-level evaluation

Abbreviation	Description	Quantity	%
1dAVb	1st degree AV block	28	3.4%
RBBB	right bundle branch block	34	4.1%
LBBB	left bundle branch block	30	3.6%
SB	sinus bradycardia	16	1.9%
AF	atrial fibrillation	13	1.6%
ST	sinus tachycardia	36	4.4%

### MCMA

**Fig. 1:**
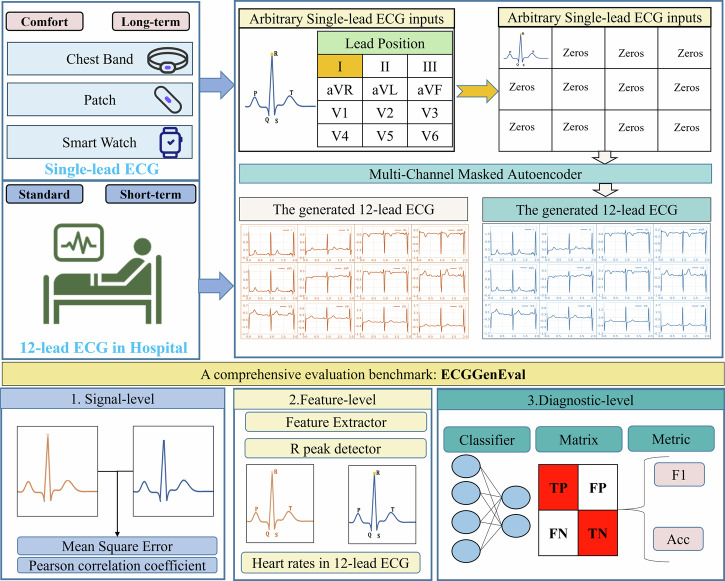
The 12-lead ECG generation with single-lead ECG. Top-left: the input single-lead ECG can be arbitrary, including I, II, III, aVR, aVL, avF, V1, V2, V3, V4, V5, V6.Top-right: it shows the detailed process, and this case takes lead I as an example. Bottom: the evalution benchmark, including signal-level, feature-level and diagnoistic-level.

Multi-Channel Masked Autoencoder (MCMA) masks 11 different leads, leaving only a single-lead ECG to generate the standard 12-lead ECG. MCMA takes a single-lead ECG as input and produces a 12-lead ECG as output, both with a signal length of 1024. The abstract of MCMA is seen in Fig. 1. In this study, no preprocessing steps like filtering or scaling are applied to avoid altering the ECG signals. Additionally, MCMA uses a multi-channel masked configuration to reduce training and inference costs, requiring only one model, which sets it apart from related approaches in the prior works^30,32,33,35^.

### Model architecture

**Fig. 2:**
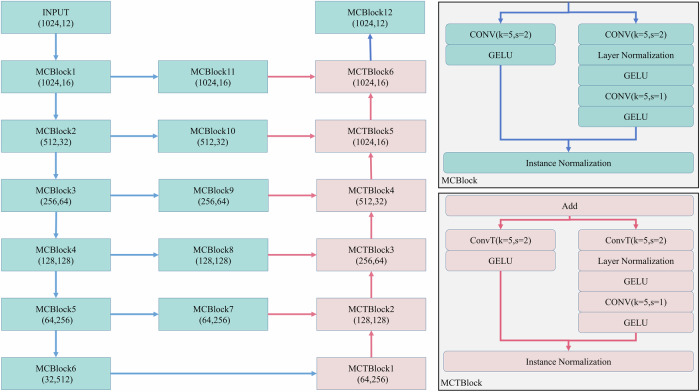
The detailed model architecture, the proposed model mainly includes MCBlock and MCTBlock. Left: the situation of each layer and shape changes from input to output. Top-right: composition of MCBlock, including two branches, which will achieve downsampling; Bottom-right: composition of MCTBlock, including two branches, which will achieve upsampling.

MCMA needs a designed architecture, as seen in Fig. 2. Motivated by ResNet^40^ and UNet^41^. The model includes two modules, namely, the downsampling and upsampling modules, which are composed of the multi-convolution block (MCBlock) and multi-convolution-transpose block (MCTBlock), respectively. The kernel size (*k*) is 5 and the window size (*s*) is 2. The choice of setting kernel size as 5 for MCBlock and MCTBlock layers aims in achieving effective feature extraction in deep learning models, particularly in those processing data with rich spatial hierarchies. The window size is usually 2 for the striding process, which can reduce the feature dimension and improve the learning ability. The activation function is GELU. The experimental results with different hyperparameters can be seen in Supplementary materials. To improve the gradient stability, layer normalization (LN) and instance normalization (IN) are used in each block. The skip connections can speed up the convergence rate of the model and improve the representation ability. Additionally, the basic training recipe is provided in Table 4.

**Table 4:** The hyperparameters configuration in the MCMA training process

hyperparameters	configuration
Batch size	256
Epochs	100
Signal Length	1024
Optimizer	Adam
Learning rate	1e-3

### MCMA implementation

#### Padding strategy

MCMA utilizes a zero-padding strategy to retain the space information for each single-lead ECG. When the single-channel ECG is processed into the 12-channel format, while the other channels are zeros, as seen in Eq. (1).

1 $$P(ec{g}_{12},i)={I}_{z}\times ec{g}_{12}[i]$$

In Eq. (1), the shape of index matrix for zero-padding is 12 × 1, *I*_*z*_(*i*) = 1 with other elements being zeros. Specifically, the output shape equals the input shape, and the shape of *ecg*_12_ is 12 × *N*, then the shape of *ecg*_12_[*i*] is 1 × *N*, so the output shape also is 12 × *N*. With zero-padding, MCMA can adaptively solve different inputs. To highlight its advantages, the 12 copies for the single-lead ECG acts as a comparison, named as the copy-padding strategy. The index matrix for copy-padding strategy, *I*_*c*_, all elements are 1. At the same time, the arbitrary input lead and the fixed lead (lead I) are compared. In addition, the 12-lead ECG is provided in model training, and the padding strategy aims to mask the original 11-lead ECG with zeros or the remaining single-lead ECG in the standard 12-lead ECG. Meanwhile, only the single-lead ECG exists in the real-world application process, it should be with the padding strategy for the proposed framework.

#### Loss function

The generative models mainly involve autoencoder(AE)^42^, generative adversarial network(GAN)^34^, diffusion model^43^. Although the diffusion model has shown its great potential and ability in various tasks, the sampling speed^44^ is challenging. GAN^32,33,35^ and AE^30^ have been studied by the previous research works. Additionally, it is worth mentioning that the traditional GAN is not enough to complete this task, which supports converting random noise into the generative signals. Therefore, the researchers of this task adopted a conditional generative adversarial network, including Seo et al.^32^ Joo et el.^33^, and our previous study^35^. In this study, the autoencoder can be a feasible solution for this 12-lead ECG reconstruction task, due to the training stability. Further, the proposed framework needs to be compared with the GAN-based^32,33,35^ and AE-based^30^ methods.

The autoencoder (*AE*) can extract the latent representation from the raw data and convert the latent representation into the target output. The common loss function (*L*) is shown in Eq. (2).

2 $$L=| | ec{g}_{12}-AE(ec{g}_{1})| {| }^{2}$$

In Eq. (2), the 12-lead and single-lead ECG signals are represented by *ecg*_12_ and *ecg*_1_. *P* means the padding strategy, as shown in Eq. (1), *i* means the index, varying from 1 to 12. MCMA employs a zero-padding strategy as default, while copy-padding is utilized for comparative analysis within the ablation study.

#### Inferencing MCMA

After the training process, MCMA can be used in real-world applications, i.e., the inferencing (testing) process. The single-lead ECG with the zeros-padding strategy is the input of MCMA. Then, the application process for MCMA can be seen in Eq. (3).

3 $${g}_{ecg}=AE({I}_{z}\times ec{g}_{1})$$

In Eq. (3), *g*_*ecg*_ is the generated 12-lead ECG with MCMA, *ecg*_1_ is the single-lead ECG collected by wearable devices, *I*_*z*_ can convert *ecg*_1_ into the input of *AE*.

### Comprehensive evaluations of ECG reconstruction

This study introduces ECGGenEval, a comprehensive evaluation benchmark for 12-lead ECG reconstruction, including three distinct dimensions: signal-level, feature-level, and diagnostic-level.

#### Signal-level evaluations

This study adopts the Pearson correlation coefficient (*PCC*) and mean square error (*MSE*) in the signal-level evaluation. The real and generated ECG signal are defined as *r*_*ecg*_ and *g*_*ecg*_. Then, the definitions for *PCC* and *MSE* are shown in Eqs. (4) and (5).

4 $$PCC({r}_{ecg},{g}_{ecg})=\frac{\mu ({r}_{ecg}\times {g}_{ecg})-\mu ({r}_{ecg})\mu ({g}_{ecg})}{\sigma ({r}_{ecg})\sigma ({g}_{ecg})}$$

5 $$MSE({r}_{ecg},{g}_{ecg})=\mu ({({r}_{ecg}-{g}_{ecg})}^{2})$$

In these equations, as Eqs. (4) and (5), *μ*(*) and *σ*(*) denotes the mean value and standard deviation, respectively. *PCC* varies from −1 to 1, and *MSE* is bigger than 0. The relationship between *PCC* and generation performance is positively related, while the relationship between *MSE* and generation performance is negatively related. For the signal-level evaluation, a better generative model should be with a higher *PCC* and lower *MSE*.

#### Feature-level evaluations

Furthermore, this study adopts the estimated heart rate of the generated 12-lead ECG for the feature-level evaluation. Since the heart rate in real 12-lead ECG signals theoretically occurs simultaneously, and the generated signals should meet this requirement. The mean heart rate (*MHR*) at the *j*th lead can be calculated, as shown in Eq. (6).

6 $$MHR(j)=\frac{60\times (n-1)}{\mathop{\sum }\nolimits_{i = 1}^{n-1}(R(i+1,j)-R(i,j))}$$

In Eq. (6), the *i*th detected R-wave in *j*th lead is denoted as *R*(*i*, *j*), and its unit is second. Therefore, *MHR* can represent the heartbeat per minute. Based on the 12 *MHR* from different 12-lead ECG, the average value *MMHR* can be computed with Eq. (7). Then, the feature-level evaluation involves standard deviation (*SD*), Range (the difference between maximum and minimum), and coefficient of variation (*CV*), expressed as *MHR*_*SD*_, *MHR*_*Range*_ and *MHR*_*CV*_, respectively. The calculation processes can be seen in Eq. (8), Eq. (9) and Eq. (10), respectively.

7 $$MMHR=\frac{1}{12}\mathop{\sum }\limits_{j=1}^{12}\left(MHR(j)\right.$$

8 $$MH{R}_{SD}=\sqrt{\frac{1}{12}\mathop{\sum }\limits_{j=1}^{12}{(MHR(j)-MMHR)}^{2}}$$

9 $$MH{R}_{Range}=\max (MHR)-\min (MHR)$$

10 $$MH{R}_{CV}=\frac{MH{R}_{SD}}{MMHR}$$

The reference estimation is completed with the original 12-lead ECG These feature-level evaluation is good if the inter-lead heart rates are consistent.

#### Diagnostic-level evaluations

This study also adopts the diagnostic-level evaluation for this 12-lead ECG reconstruction task. MCMA is able to convert the limit-lead (even single-lead) ECG into 12-lead ECG, which bridges the limited-lead ECG to the classifiers which trained with 12-lead ECG as input. Therefore, this study can evaluate the generated 12-lead ECG using classification performance, including the precision (*Pre*), recall (*Rec*), specificity (*Spe*) and F1-score (*F*_1_), as shown in literature^5^. These calculation process of classification metric are seen in Eq. (11), Eq. (12), Eq. (13) and Eq. (14).

11 $$Pre=\frac{TP}{TP+FP}$$

12 $$Rec=\frac{TP}{TP+FN}$$

13 $$Spe=\frac{TN}{TN+FP}$$

14 $${F}_{1}=\frac{2\times TP}{2\times TP+FN+FP}$$

Also, the original classification performance with the real 12-lead ECG is the standard reference, and the generated 12-lead ECG with the other methods^30,32,33,35^ are used in the result comparison.

## Results

### Signal-level performance

First of all, the signal-level evaluation is the primary evaluation metric, such as *MSE* and *PCC*. In contrast to conventional approaches, this scheme offers a distinct advantage: it enables the conversion of an arbitrary single-lead ECG to a 12-lead ECG without the necessity of training multiple generative models. The experimental results of *MSE* and *PCC* are shown in Table 5, where the horizontal direction represents the output and the vertical direction represents the input. Besides, the reconstruction performance in the external dataset, CPSC2018, is seen in Table 6.

**Table 5:** The signal-level evaluation of mean square error (*MSE*) and Pearson correlation coefficient (*PCC*) between the generated and real 12-lead ECG in the internal testing set, PTB-XL

Output	I	II	III	aVR	aVL	aVF	V1	V2	V3	V4	V5	V6	Mean
Input													
	*MSE*												
I	0.0032	0.0095	0.0120	0.0032	0.0055	0.0101	0.0149	0.0466	0.0444	0.0293	0.0193	0.0140	0.0177
II	0.0074	0.0035	0.0112	0.0029	0.0087	0.0054	0.0166	0.0484	0.0480	0.0297	0.0183	0.0132	0.0178
III	0.0069	0.0075	0.0065	0.0056	0.0050	0.0052	0.0171	0.0497	0.0530	0.0381	0.0247	0.0167	0.0197
aVR	0.0052	0.0056	0.0162	0.0018	0.0089	0.0093	0.0150	0.0460	0.0440	0.0270	0.0158	0.0116	0.0172
aVL	0.0046	0.0090	0.0078	0.0049	0.0040	0.0072	0.0160	0.0472	0.0482	0.0343	0.0228	0.0157	0.0185
aVF	0.0077	0.0048	0.0077	0.0045	0.0067	0.0042	0.0170	0.0496	0.0514	0.0346	0.0220	0.0153	0.0188
V1	0.0072	0.0098	0.0173	0.0047	0.0093	0.0113	0.0092	0.0371	0.0464	0.0368	0.0231	0.0153	0.0190
V2	0.0085	0.0103	0.0169	0.0055	0.0100	0.0113	0.0131	0.0206	0.0288	0.0336	0.0258	0.0174	0.0168
V3	0.0080	0.0103	0.0179	0.0052	0.0099	0.0116	0.0152	0.0304	0.0172	0.0229	0.0221	0.0166	0.0156
V4	0.0074	0.0089	0.0162	0.0044	0.0096	0.0104	0.0166	0.0419	0.0293	0.0127	0.0151	0.0140	0.0156
V5	0.0067	0.0075	0.0160	0.0035	0.0093	0.0096	0.0162	0.0464	0.0402	0.0195	0.0094	0.0103	0.0162
V6	0.0065	0.0071	0.0160	0.0033	0.0092	0.0093	0.0155	0.0474	0.0452	0.0247	0.0123	0.0081	0.0171
Mean	0.0066	0.0078	0.0135	0.0041	0.0080	0.0087	0.0152	0.0426	0.0413	0.0286	0.0192	0.0140	0.0175
	*PCC*												
I	0.9880	0.7719	0.5516	0.9222	0.8357	0.5436	0.8400	0.7413	0.7410	0.8195	0.8604	0.8720	0.7906
II	0.8389	0.9870	0.6201	0.9343	0.6236	0.8670	0.8113	0.7212	0.7117	0.8219	0.8768	0.8920	0.8088
III	0.8438	0.8174	0.9778	0.8203	0.8808	0.8814	0.7995	0.7090	0.6621	0.7389	0.7965	0.8203	0.8123
aVR	0.9131	0.9030	0.3281	0.9885	0.6174	0.6038	0.8406	0.7418	0.7472	0.8443	0.9026	0.9176	0.7790
aVL	0.9249	0.7711	0.8622	0.8483	0.9763	0.7171	0.8204	0.7343	0.7037	0.7709	0.8178	0.8361	0.8152
aVF	0.8231	0.9245	0.8697	0.8637	0.7453	0.9783	0.8003	0.7083	0.6763	0.7710	0.8282	0.8486	0.8198
V1	0.8353	0.7498	0.2492	0.8562	0.5706	0.4704	0.9798	0.8071	0.7012	0.7482	0.8113	0.8406	0.7183
V2	0.8040	0.7300	0.2865	0.8249	0.5582	0.4720	0.8810	0.9830	0.8666	0.7785	0.7867	0.8070	0.7315
V3	0.8152	0.7365	0.2303	0.8351	0.5469	0.4682	0.8358	0.8842	0.9866	0.8824	0.8313	0.8251	0.7398
V4	0.8364	0.7881	0.3247	0.8698	0.5579	0.5469	0.8092	0.7784	0.8703	0.9872	0.9157	0.8752	0.7633
V5	0.8624	0.8344	0.3373	0.9096	0.5790	0.5879	0.8183	0.7408	0.7796	0.9199	0.9865	0.9471	0.7752
V6	0.8685	0.8490	0.3353	0.9202	0.5852	0.6041	0.8324	0.7329	0.7397	0.8707	0.9509	0.9871	0.7730
Mean	0.8628	0.8219	0.4977	0.8828	0.6731	0.6451	0.8391	0.7735	0.7655	0.8294	0.8637	0.8724	0.7772

**Table 6:** The signal-level evaluation of mean square error (*MSE*) and Pearson correlation coefficient (*PCC*) between the generated and real 12-lead ECG in an external testing set, CPSC2018

Output	I	II	III	aVR	aVL	aVF	V1	V2	V3	V4	V5	V6	Mean
Input													
	*MSE*												
I	0.0252	0.0335	0.0353	0.0271	0.0268	0.0330	0.0566	0.0909	0.1008	0.1018	0.117	0.1378	0.0655
II	0.0300	0.0264	0.0330	0.0265	0.0309	0.0271	0.0613	0.0971	0.1059	0.1031	0.1173	0.1379	0.0664
III	0.0304	0.0305	0.0273	0.029	0.0263	0.0270	0.0605	0.0980	0.1125	0.1138	0.1267	0.1434	0.0688
aVR	0.0290	0.0299	0.0389	0.0233	0.0318	0.0314	0.0564	0.0943	0.1023	0.1007	0.1157	0.1371	0.0659
aVL	0.0276	0.0334	0.0294	0.0294	0.0244	0.0298	0.0585	0.0929	0.1080	0.1128	0.1261	0.1432	0.0680
aVF	0.0311	0.0277	0.0287	0.0282	0.0286	0.0260	0.0631	0.0996	0.1110	0.1102	0.1235	0.1416	0.0683
V1	0.0310	0.0346	0.0394	0.0278	0.0317	0.0338	0.0443	0.0797	0.1038	0.1128	0.1264	0.1432	0.0674
V2	0.0311	0.0342	0.0385	0.0293	0.0315	0.0335	0.0533	0.0540	0.0792	0.1049	0.1271	0.1447	0.0635
V3	0.0307	0.0336	0.0397	0.0289	0.0319	0.0333	0.0567	0.0671	0.0634	0.0903	0.1202	0.1420	0.0615
V4	0.0302	0.0324	0.0398	0.0278	0.0320	0.0327	0.0593	0.0832	0.0787	0.0764	0.1111	0.1381	0.0618
V5	0.0294	0.0319	0.0397	0.0271	0.0318	0.0327	0.0589	0.0910	0.0933	0.0884	0.0979	0.1331	0.0629
V6	0.0292	0.0319	0.0396	0.0268	0.0317	0.0327	0.0586	0.0937	0.1008	0.0960	0.1087	0.1226	0.0644
Mean	0.0296	0.0317	0.0358	0.0276	0.0300	0.0311	0.0573	0.0868	0.0967	0.1009	0.1181	0.1387	0.0654
	*PCC*												
I	0.9822	0.7718	0.4308	0.9040	0.7185	0.5413	0.7310	0.6619	0.7080	0.8190	0.8646	0.8728	0.7505
II	0.7849	0.9858	0.5285	0.9320	0.4233	0.8764	0.6558	0.5909	0.6583	0.8081	0.8613	0.8670	0.7477
III	0.7786	0.8345	0.9732	0.8135	0.8331	0.8918	0.6635	0.5894	0.6015	0.7198	0.7714	0.7889	0.7716
aVR	0.8778	0.9155	0.2331	0.9864	0.4149	0.6631	0.7175	0.6303	0.7002	0.8423	0.8963	0.9087	0.7322
aVL	0.8600	0.7544	0.8151	0.8048	0.9665	0.7007	0.6989	0.6380	0.6387	0.7290	0.7782	0.7947	0.7649
aVF	0.7502	0.9300	0.8495	0.8552	0.6167	0.9787	0.6224	0.5646	0.6130	0.7476	0.7996	0.8123	0.7616
V1	0.7722	0.7332	0.1848	0.8240	0.3818	0.4911	0.9668	0.7416	0.6645	0.7282	0.7736	0.7942	0.6713
V2	0.7528	0.7210	0.2052	0.8028	0.4038	0.4711	0.7921	0.9793	0.8633	0.7866	0.7740	0.7766	0.6940
V3	0.7674	0.7514	0.1378	0.8246	0.3714	0.5053	0.7275	0.8684	0.9828	0.9008	0.8386	0.8174	0.7078
V4	0.7865	0.7963	0.1515	0.8625	0.3542	0.5504	0.6811	0.7256	0.8777	0.9856	0.9178	0.8726	0.7135
V5	0.8210	0.8172	0.1728	0.8988	0.3734	0.5576	0.6929	0.6617	0.7665	0.9207	0.9848	0.9393	0.7172
V6	0.8356	0.8165	0.1877	0.9088	0.3837	0.5641	0.7024	0.6372	0.7102	0.8664	0.9429	0.9859	0.7118
Mean	0.8141	0.8190	0.4058	0.8681	0.5201	0.6493	0.7210	0.6907	0.7321	0.8212	0.8503	0.8525	0.7287

### Feature-level performance

This study also provides the feature-level evaluation results for MCMA, including the standard deviation *MHR*_*SD*_, Range *MHR*_*Range*_ and coefficient of variation *MHR*_*CV*_. The feature-level evaluation results in the internal testing set PTB-XL and external testing set CPSC2018 are shown in Table 7 and Table 8, respectively. In the mentioned two tables, the first group is the reference value of the original 12-lead ECG. Additionally, the R-peak recognition is completed by algorithm^45^.

**Table 7:** The feature-level evaluation for the generated and real 12-lead ECG in the internal testing dataset, PTBXL

Metric	*MHR*_*SD*_	*MHR*_*CV*_	*MHR*_*Range*_
Input			
Original	2.2137	3.21%	7.2195
I	1.1164	1.63%	3.5415
II	1.0073	1.51%	3.0958
III	1.2042	1.80%	3.7227
aVR	1.0785	1.61%	3.3192
aVL	1.1893	1.77%	3.6891
aVF	0.934	1.40%	2.8122
V1	0.9944	1.51%	3.1111
V2	0.9582	1.48%	3.0753
V3	1.0777	1.66%	3.4607
V4	0.9287	1.43%	2.9728
V5	1.0264	1.56%	3.2796
V6	1.0627	1.61%	3.3682
Mean	1.0481	1.58%	3.2874

**Table 8:** The feature-level evaluation for the generated and real 12-lead ECG in the external testing dataset, CPSC2018

Metric	*MHR*_*SD*_	*MHR*_*CV*_	*MHR*_*Range*_
Input			
Original	2.1313	2.65%	7.1267
I	1.3510	1.70%	4.4984
II	0.7732	0.99%	2.4776
III	0.9133	1.17%	2.9432
aVR	0.8590	1.10%	2.7977
aVL	1.3069	1.69%	4.2717
aVF	0.8146	1.04%	2.5931
V1	0.9355	1.24%	3.0275
V2	0.8629	1.16%	2.8255
V3	0.8467	1.16%	2.7630
V4	0.8529	1.14%	2.7957
V5	0.9544	1.28%	3.1345
V6	0.9093	1.22%	2.9712
Mean	0.9483	1.24%	3.0916

### Diagnostic-level performance

Lastly, this study demonstrates the diagnostic-level performance of MCMA. The classifier is trained and validated by Ribeiro et al.^5^, which only accepts the 12-lead ECG. Then, it is essential to present the classification performance with the generated 12-lead ECG. For example, Table 9 shows the classification performance of the generated 12-lead ECG with lead I. The detailed diagnostic-level evaluations are shown in Table 10, including the original 12-lead ECG (as the reference), the single-lead ECG (i.e., MCMA input) and the generated 12-lead ECG (i.e., MCMA output), which directly shows the gain in the arrhythmia classification task.

**Table 9:** The diagnostic-level evaluation for MCMA, as the generated 12-lead ECG is from lead I ECG, CODE-test

Metric	*Pre*	*Rec*	*Spe*	*F*_1_
Class				
1dAVb	0.8750	0.7500	0.9962	0.8077
RBBB	0.8788	0.8529	0.9950	0.8657
LBBB	0.9630	0.8667	0.9987	0.9123
SB	0.7273	1.0000	0.9926	0.8421
AF	0.5833	0.5385	0.9939	0.5600
ST	0.9459	0.9459	0.9975	0.9459
Mean	0.8289	0.8257	0.9956	0.8223

**Table 10:** The diagnostic-level evaluation for the generated 12-lead ECG in another external testing dataset, CODE-test

Metric	*Pre*	*Rec*	*Spe*	*F*_1_
Input				
Original 12-lead^5^	0.8747	0.9100	0.9958	0.8872
I	0.3971	0.1309	0.9910	0.1824
MCMA+I	0.8289	0.8257	0.9956	0.8223
MCMA GAIN	0.4318	0.6948	0.0046	0.6399
II	0.0682	0.0339	0.9778	0.0333
MCMA+II	0.8401	0.8588	0.9946	0.8410
MCMA GAIN	0.7719	0.8249	0.0168	0.8077
III	0.1667	0.0056	0.9998	0.0108
MCMA+I	0.7237	0.6784	0.9923	0.6840
MCMA GAIN	0.5570	0.6728	−0.0075	0.6732
aVR	0.0000	0.0000	0.9985	0.0000
MCMA+aVR	0.4775	0.4261	0.9816	0.4348
MCMA GAIN	0.4775	0.4261	−0.0169	0.4348
aVL	0.0000	0.0000	0.9998	0.0000
MCMA+aVR	0.5728	0.6390	0.9827	0.5905
MCMA GAIN	0.5728	0.6390	−0.0171	0.5905
aVF	0.0000	0.0000	1.0000	0.0000
MCMA+aVR	0.5226	0.6532	0.9706	0.5223
MCMA GAIN	0.5226	0.6532	−0.0294	0.5223
V1	0.2641	0.2510	0.9973	0.2573
MCMA+V1	0.7670	0.8776	0.9923	0.8146
MCMA GAIN	0.5029	0.6266	−0.0050	0.5573
V2	0.1667	0.0611	1.0000	0.0894
MCMA+V2	0.7377	0.8435	0.9915	0.7824
MCMA GAIN	0.5710	0.7824	−0.0085	0.6930
V3	0.2428	0.1267	0.9990	0.1469
MCMA+V3	0.7669	0.8438	0.9929	0.8006
MCMA GAIN	0.5241	0.7171	−0.0061	0.6537
V4	0.1667	0.0090	1.0000	0.0171
MCMA+V4	0.7943	0.8373	0.9936	0.8090
MCMA GAIN	0.6276	0.8283	−0.0064	0.7919
V5	0.0000	0.0000	1.0000	0.0000
MCMA+V5	0.7582	0.8285	0.9917	0.7854
MCMA GAIN	0.7582	0.8285	−0.0083	0.7854
V6	0.0833	0.0049	0.9996	0.0093
MCMA+V6	0.7450	0.8113	0.9921	0.7717
MCMA GAIN	0.6617	0.8064	−0.0075	0.7624

### Comparison with other methods

MCMA compares with other research works, including Garg et al.^30^, Seo et al.^32^, and Joo et al.^33^. As known, Garg et al.^30^ adopt the lead II, while Seo et al.^32^ and Joo et al.^33^ utilizes the lead I. Moreover, MCMA can convert arbitrary single-lead ECG into the standard 12-lead ECG. The comparisons in signal-level, feature-level, and diagnostic-level are shown in Table 11, Table 12, and Table 13.

**Table 11:** The signal-level comparison of different methods in PTB-XL and CPSC2018

Dataset	Metric	Method	Input	I	II	III	aVR	aVL	aVF	V1	V2	V3	V4	V5	V6	Mean
PTB-XL	*MSE*	Garg et al.^30^	II	0.0122	0.0001	0.0123	0.0031	0.0122	0.0031	0.0323	0.0690	0.0735	0.0477	0.0374	0.0474	0.0292
		MCMA	II	0.0074	0.0035	0.0112	0.0029	0.0087	0.0054	0.0166	0.0484	0.0480	0.0297	0.0183	0.0132	0.0178
		Seo et al.^32^	I	0.0001	0.0148	0.0149	0.0037	0.0038	0.0148	0.0263	0.0579	0.0640	0.0477	0.0388	0.0482	0.0279
		Joo et al.^33^	I	0.0002	0.0189	0.0187	0.0050	0.0059	0.0190	0.0317	0.0868	0.0920	0.0686	0.0517	0.0546	0.0378
		Zhan et al.^35^	I	0.0003	0.0153	0.0154	0.0041	0.0042	0.0153	0.0267	0.0589	0.0660	0.0495	0.0391	0.0483	0.0286
		MCMA	I	0.0032	0.0095	0.0120	0.0032	0.0055	0.0101	0.0149	0.0466	0.0444	0.0293	0.0193	0.0140	0.0177
	*PCC*	Garg et al.^30^	II	0.8087	0.9986	0.6409	0.9330	0.6029	0.8873	0.7792	0.7344	0.7056	0.7959	0.8482	0.8427	0.7981
		MCMA	II	0.8389	0.9870	0.6201	0.9343	0.6236	0.8670	0.8113	0.7212	0.7117	0.8219	0.8768	0.8920	0.8088
		Seo et al.^32^	I	0.9983	0.7527	0.5849	0.9189	0.8576	0.5407	0.8237	0.7805	0.7472	0.7931	0.8346	0.8295	0.7885
		Joo et al.^33^	I	0.9962	0.6807	0.4571	0.8905	0.8178	0.4021	0.7827	0.6928	0.6416	0.7112	0.7837	0.7827	0.7199
		Zhan et al.^35^	I	0.9923	0.7332	0.5673	0.9093	0.8390	0.5182	0.8172	0.7811	0.7415	0.7818	0.8290	0.8246	0.7779
		MCMA	I	0.9880	0.7719	0.5516	0.9222	0.8357	0.5436	0.8400	0.7413	0.7410	0.8195	0.8604	0.8720	0.7906
CPSC2018	*MSE*	Garg et al.^30^	II	0.0215	0.0024	0.0289	0.0668	0.0306	0.0101	0.0958	0.1232	0.1532	0.1651	0.2047	0.2579	0.0967
		MCMA	II	0.0300	0.0264	0.0330	0.0265	0.0309	0.0271	0.0613	0.0971	0.1059	0.1031	0.1173	0.1379	0.0664
		Seo et al.^32^	I	0.0036	0.0315	0.0454	0.0408	0.0162	0.0339	0.0861	0.1171	0.1488	0.1694	0.2084	0.2655	0.0972
		Joo et al.^33^	I	0.0108	0.0409	0.0455	0.0371	0.0228	0.0428	0.0993	0.1834	0.1827	0.1821	0.2185	0.2755	0.1118
		Zhan et al.^35^	I	0.0141	0.0393	0.0443	0.0304	0.0237	0.0403	0.0838	0.1225	0.1571	0.1759	0.2149	0.2704	0.1014
		MCMA	I	0.0252	0.0335	0.0353	0.0271	0.0268	0.0330	0.0566	0.0909	0.1008	0.1018	0.1170	0.1378	0.0655
	*PCC*	Garg et al.^30^	II	0.7552	0.9981	0.5686	0.9264	0.4242	0.8901	0.6225	0.5803	0.6329	0.7861	0.8376	0.8369	0.7382
		MCMA	II	0.7849	0.9858	0.5285	0.9320	0.4233	0.8764	0.6558	0.5909	0.6583	0.8081	0.8613	0.8670	0.7477
		Seo et al.^32^	I	0.9978	0.7402	0.4153	0.8932	0.7442	0.4830	0.7129	0.6268	0.6619	0.7828	0.8378	0.8382	0.7278
		Joo et al.^33^	I	0.9950	0.7303	0.3517	0.8827	0.7181	0.4701	0.6453	0.5385	0.5238	0.7481	0.8104	0.8002	0.6845
		Zhan et al.^35^	I	0.9889	0.7246	0.4069	0.8845	0.7351	0.4805	0.7071	0.6475	0.6538	0.7636	0.8313	0.8274	0.7209
		MCMA	I	0.9822	0.7718	0.4308	0.9040	0.7185	0.5413	0.7310	0.6619	0.7080	0.8190	0.8646	0.8728	0.7505

**Table 12:** The feature-level comparison of different methods in PTB-XL and CPSC2018

Dataset	Method	Input	*MHR*_*SD*_	*MHR*_*CV*_	*MHR*_*Range*_
PTB-XL	Original	*	2.2137	3.21%	7.2195
	Garg et al.^30^	Lead II	1.1608	1.70%	3.5872
	MCMA	Lead II	1.0073	1.51%	3.0958
	Seo et al.^32^	Lead I	1.8943	2.74%	6.3984
	Joo et al.^33^	Lead I	2.6891	4.03%	8.8273
	Zhan et al.^35^	Lead I	2.6952	3.82%	9.0689
	MCMA	Lead I	1.1164	1.63%	3.5413
CPSC2018	Original	*	2.1313	2.65%	7.1267
	Garg et al.^30^	Lead II	0.9545	1.24%	3.0523
	MCMA	Lead II	0.7732	0.99%	2.4776
	Seo et al.^32^	Lead I	2.1899	2.79%	7.5269
	Joo et al.^33^	Lead I	2.4136	3.31%	8.1059
	Zhan et al.^35^	Lead I	2.8610	3.71%	9.9589
	MCMA	Lead I	1.3510	1.40%	4.4984

**Table 13:** The diagnostic-level comparison of different methods in CODE-test

Method	Input	*Pre*	*Rec*	*Spe*	*F*_1_
Reference	12-lead ECG	0.8747	0.9100	0.9958	0.8872
Garg et al.^30^	Lead II	0.7268	0.8542	0.9881	0.7808
Input for MCMA	Lead II	0.0682	0.0339	0.9778	0.0333
MCMA	Lead II	0.8401	0.8588	0.9946	0.8410
Seo et al.^32^	Lead I	0.8248	0.8480	0.9948	0.8299
Joo et al.^33^	Lead I	0.7817	0.7846	0.9938	0.7730
Zhan et al.^35^	Lead I	0.8171	0.8739	0.9946	0.8423
Input for MCMA	Lead I	0.3971	0.1309	0.9910	0.1824
MCMA	Lead I	0.8289	0.8257	0.9956	0.8223

### Ablation study

MCMA utilizes two key modules, one for arbitrary single-lead ECG reconstruction, and another for zero-padding strategy. Then, it is necessary to compare with different settings, including fixed-channel (lead I as an example) and copy-padding strategy. The signal-level evaluation metric includes mean square error (*MSE*) and Pearson correlation coefficient (*PCC*). The experimental results comparison with different settings can be shown in Table 14, including the lead I and the average value for 12 single-lead ECG. In most cases, MCMA has achieved excellent result in 12-lead ECG reconstruction task.

**Table 14:** The ablation study for the proposed framework, MCMA, which adopts the zero-padding strategy and supports arbitrary single-lead ECG as input

Setting			PTB-XL		CPSC2018	
Arbitrary	Padding	Input	*MSE*	*PCC*	*MSE*	*PCC*
No	Zeros	Lead I	**0.0176**	0.7879	0.0659	0.7480
Yes	Copy	Lead I	0.0183	0.7608	0.0674	0.6885
Yes	Zeros	Lead I	0.0177	0.7906	0.0655	0.7505
No	Zeros	12 Single-lead	0.0406	0.3310	0.0911	0.2956
Yes	Copy	12 Single-lead	0.0197	0.7198	0.0692	0.6291
Yes	Zeros	12 Single-lead	0.0175	0.7772	0.0654	0.7287

### Case study

**Fig. 3:**
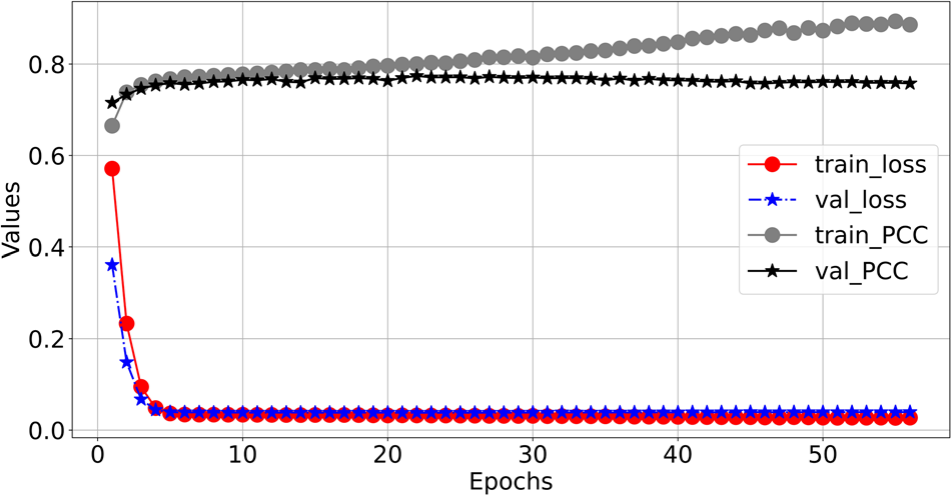
The mean square error and Pearson correlation coefficient in the training process. The red circle means training loss, the blue star means validation loss, the black circle means training Pearson correlation coefficient (PCC), and the black star means validation Pearson correlation coefficient (PCC).

**Fig. 4:**
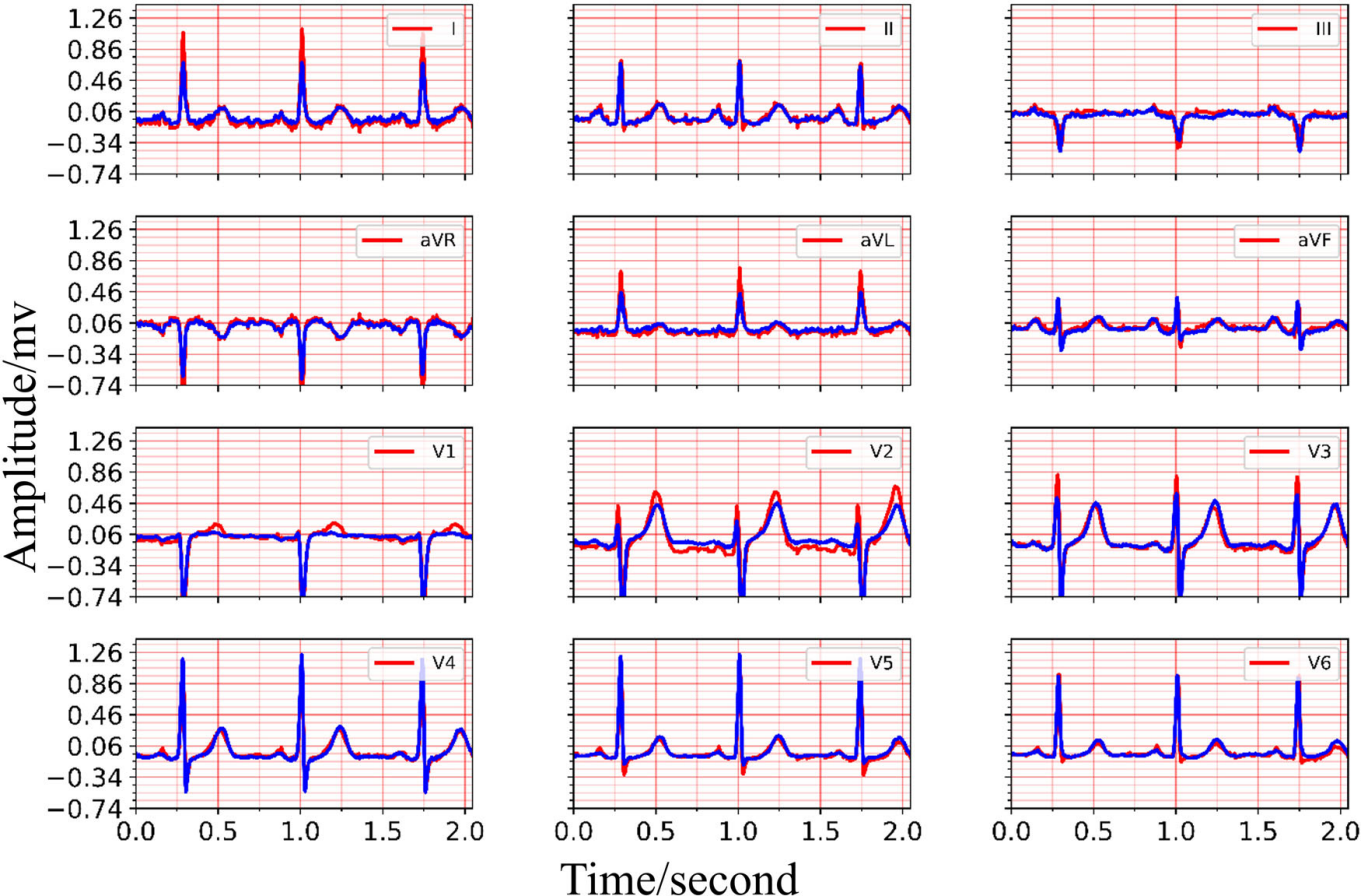
The 12-lead ECG reconstruction performance in the internal testing set PTB-XL, the red lines are the real signals while the blue lines represent the generated signals. Top: the real and reconstructed signal of lead I, lead II, lead III. Middle: the real and reconstructed signal of lead aVR, lead aVL, lead avF, lead V1, lead V2, lead V3. Bottom: the real and reconstructed signal of lead V4, lead V5, lead V6.

**Fig. 5:**
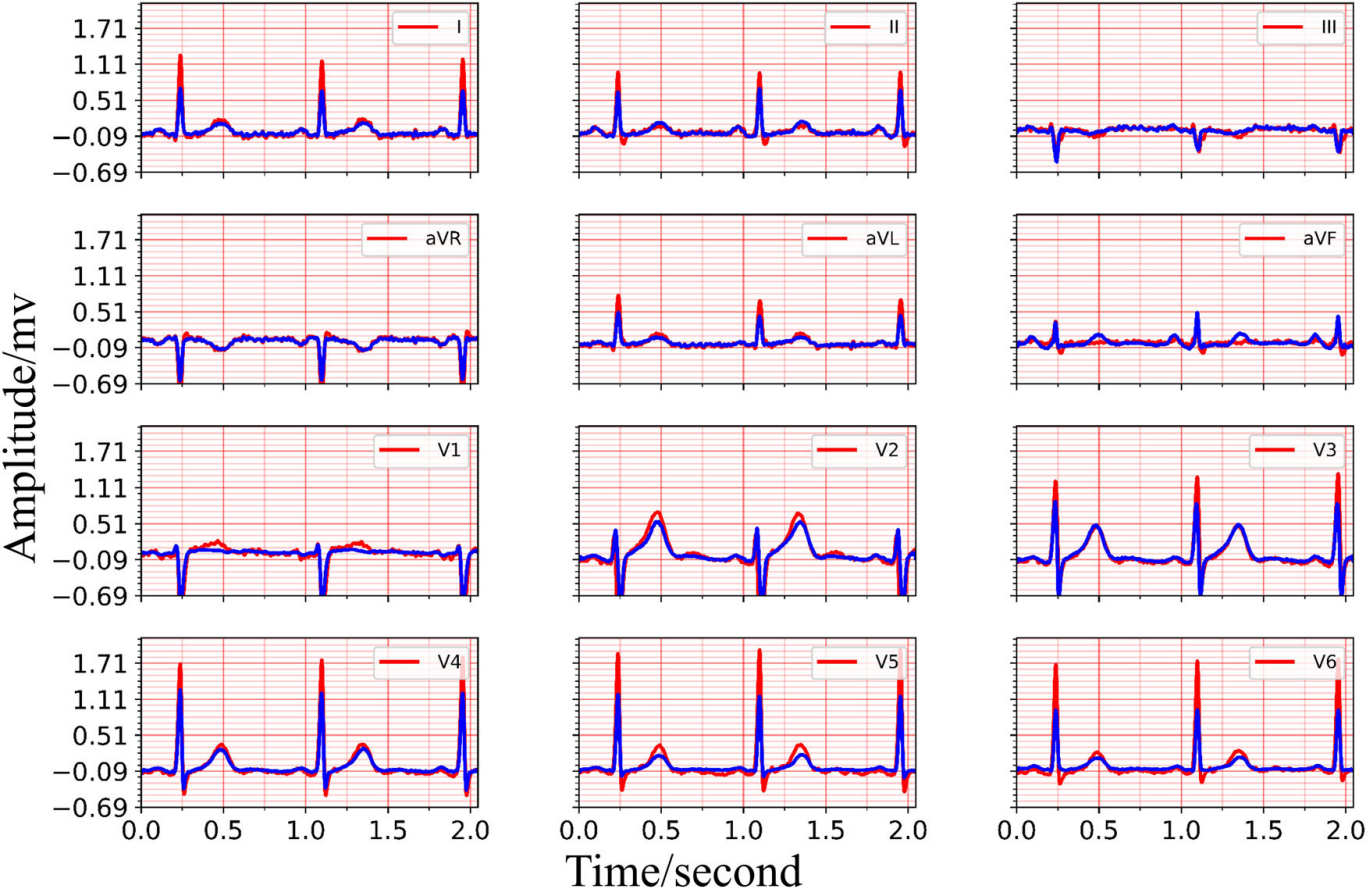
The 12-lead ECG reconstruction performance in the external testing set CPSC2018, the red lines are the real signals while the blue lines represent the generated signals. Top: the real and reconstructed signal of lead I, lead II, lead III. Middle: the real and reconstructed signal of lead aVR, lead aVL, lead avF, lead V1, lead V2, lead V3. Bottom: the real and reconstructed signal of lead V4, lead V5, lead V6.

The training process details of MCMA can be illustrated as seen in Fig. 3. To show the advantages of the proposed framework, the generated and real 12-lead ECG should be clearly shown in Fig. 4, in which the generated and the real signals are colored blue and red. Figure 4 demonstrates the great generation ability of the proposed framework. For example, the average *MSE* and *PCC* between the generated and real 12-lead ECG is 0.0032 and 0.9560, and it is concluded that the generator can generate 12-lead ECG with single-lead ECG. Besides the internal testing dataset (i.e., PTB-XL), the external testing dataset’s (i.e., CPSC2018) reconstruction performance demonstrates the proposed framework’s advantages from another aspect, as seen in Fig. 5.

**Fig. 6:**
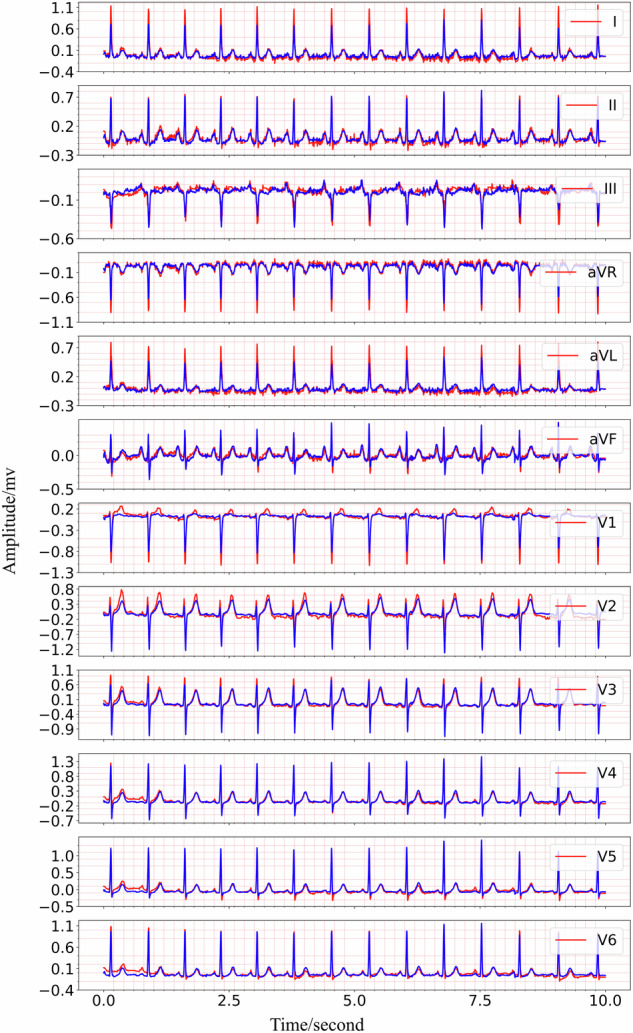
The generated and real 10-s 12-lead ECG, demonstrating its advantages for variable-duration ECG reconstruction, the red lines are the real signals while the blue lines represent the generated signals. From the top to the bottom, the signals are lead I, lead II, lead III, lead aVR, lead aVL, lead avF, lead V1, lead V2, lead V3, lead V4, lead V5, lead V6.

Based on the experimental result provided in Fig. 4 and Fig. 5, it is shown that the multi-channel masked autoencoder (MCMA) can be used to reconstruct the 12-lead ECG with single-lead ECG. In clinical practice, the ECG collected by wearable devices can be with different signal length, instead of the fixed length. It is necessary to demonstrate the proposed framework cloud also works with the variable-duration ECG signals, and the signal reconstruction result with 10-s ECG is seen in Fig. 6. In this case, the 5000 points should be filled with the extra 120 points, and it can be as the 5 individual samples for MCMA to reconstruct 12-lead ECG with single-lead ECG as input.

## Discussion

In this work, we propose a multi-channel masked autoencoder, MCMA, for generating the standard 12-lead ECG with arbitrary single-lead ECG. Further, this study establishes a comprehensive evaluation benchmark, ECGGenEval, including the signal-level, feature-level, and diagnostic-level evaluation. MCMA can work well in ECGGenEval, achieving state-of-the-art performance. MCMA can convert arbitrary single-lead ECG into 12-lead ECG, instead of the fixed-lead ECG^30,32,33,35^. Secondly, we provide multiple-level evaluation results in an internal and two external testing datasets, and the details are as follows.

Firstly, according to the signal-level evaluation results from Tables 5 and 6, on the mentioned experimental results, it is known that the proposed framework can reconstruct high-fidelity 12-lead ECG with single-lead ECG. The average *MSE* and *PCC* in PTB-XL are 0.0175 and 0.7772, while the average *MSE* and *PCC* in CPSC2018 are 0.0654 and 0.7287, respectively. The reconstruction performance in the internal and external testing dataset can demonstrate its advantages, and MCMA can reconstruct the standard 12-lead ECG with arbitrary single-lead ECG as input. Therefore, the proposed method can provide a feasible solution when collecting the standard 12-lead ECG is inconvenient and difficult, like remote cardiac healthcare. In the signal-level comparison, the *MSE* and *PCC* for generating 12-lead ECG with lead II are 0.0178 and 0.8088, better than Garg et al.^30^, with the *MSE* of 0.0292 and *PCC* of 0.7981. Therefore, MCMA can be used for 12-lead ECG reconstruction tasks while the single-lead ECG is collected, and the signal-level evaluation provides a novel solution in real-world cardiac healthcare applications.

Secondly, Tables 7 and 8 complete the feature-level evaluation. For the internal testing dataset, PTB-XL, Table 7 demonstrates that the heart rate estimation in different leads is similar in the generated 12-lead ECG, and it is even better than the original 12-lead ECG. The estimated heart rate from the real 12-lead ECG may be different, since the noise exists in special channels. Table 7 shows that the average *MHR*_*SD*_, *MHR*_*CV*_, and *MHR*_*Range*_ are 1.0481, 1.58%, and 3.2874, in which the optimal result is from the generated 12-lead ECG by lead V4 ECG. Table 8 shows the external evaluation in CPSC2018, the average *MHR*_*SD*_, *MHR*_*CV*_ and *MHR*_*Range*_ are 0.9483, 1.24%, and 3.0916, while the optimal result is from the generated 12-lead ECG by lead II ECG. The generated 12-lead ECG from arbitrary single-lead ECG can produce a good heart rate consistency in different leads, and it can even be better than the original 12-lead ECG in some cases, due to the ECG signal denoising function in the proposed framework. Table 12 demonstrates the advantages of MCMA over others, which can be highlighted as red. Therefore, the feature-level evaluation can demonstrate the advantages of MCMA.

Based on Table 9, the classifier can adopt the generated 12-lead ECG for arrhythmia classification. The average F1-score over 6 classes is 0.8319. Then, it is proven that MCMA can convert the single-lead ECG into the 12-lead ECG, and the generated 12-lead ECG can retain the pathological information, and it is different to the signal-level and feature-level evaluation. Therefore, with the multi-channel masked autoencoder, it is possible to complete arrhythmia classification with single-lead ECG, like lead I ECG in Table 9. Further, according to Table 10, the classification performance of the generated 12-lead ECG is better than that of single-lead ECG and similar to the real 12-lead ECG, which can demonstrate the classification performance gain brought by MCMA. The generated 12-lead from lead I can provide the closest classification performance, the average *F*_1_ is 0.8319, which exceeds other cases. According to Table 13, the classification performance with generated 12-lead ECG is improved. For example, taking lead II as input, Garg et al.^30^can achieve an *F*_1_ of 0.7807, lower than the proposed method. Similarly, with the lead I as input, Seo et al.^32^ and Joo et al.^33^ have an *F*_1_ of 0.8299 and 0.7730, respectively, while MCMA can be with a *F*_1_ with 0.8223. From the view of classification task, the classification performance in the above tables demonstrates the generated 12-lead ECG can be used for cardiac abnormality detection, which can prove its advantage in bridging the single-lead ECG and 12-lead ECG, and it is effective to generate the pathological information with single-lead ECG as input.

As Table 14 shows, the proposed framework is effective. The multi-channel strategy can support arbitrary single-lead to generate 12-lead ECG. Although the reconstruction performance of lead I is slightly lower than the fixed-channel. When the lead I ECG inputs, the fixed-channel can have a *MSE* of 0.0176 and a *PCC* of 0.7879, while MCMA can be with a *MSE* of 0.0177 and a *PCC* of 0.7906. However, for the fixed-channel, it is difficult to realize 12-lead ECG reconstruction with other leads, and the training and inference cost is largely different in training and storing 12 models with this setting. Further, the zero-padding strategy is better than the copy-padding strategy, while the two strategies both support the 12-lead reconstruction with arbitrary single-lead ECG. The mean *MSE* and *PCC* in MCMA are 0.0175 and 0.7772, while the mean *MSE* and *PCC* in copy-padding are 0.0197 and 0.7198, respectively.

This study is with the following advantages, from the engineering and clinical perspectives. Firstly, the generated signal is similar to the original signal, as the mean square errors of 0.0175 and 0.0654, correlation coefficients of 0.7772 and 0.7287 in the signal-level evaluation. Secondly, the generated signal can be used in the arrhythmia classification, as the average *F*_1_ with two generated 12-lead ECG is 0.8233 and 0.8410 in the diagnostic-level evaluation. According to the mentioned advantages, the contributions are as follows:

Further, this study is expected to be a feasible solution for wearable ECG monitoring, and it is able to improve the clinical importance of arbitrary single-lead ECG. For this research project, these experimented is conducted in these public datasets, such as PTB-XL and CPSC2018. Naturally, there are some limitations in this study, and these issues should be addressed in the future, as follows. High-quality electrocardiogram (ECG) signal acquisition method can significantly impact the reconstruction performance, and it may be addressed in the sensing layer^46^ or the algorithmic layer^43^. The generated signals necessitate evaluation by professional clinicians to ascertain their viability as a long-term substitute for the conventional 12-lead ECG in continuous monitoring scenarios.In other words, the question is whether a physician can render an equivalent diagnosis utilizing the 12-lead ECG generated by MCMA. Consequently, additional research endeavors are essential to advance the mentioned problems, ultimately realizing the considerable clinical relevance and practical utility.

In a word, this study proposes a novel generative framework to reconstruct 12-lead ECG with a single-lead ECG, as multi-channel masked autoencoder (MCMA), and it involves two main contributions. Firstly, unlike other methods, the proposed framework can convert arbitrary single-lead ECG into the standard 12-lead ECG. The experimental results showed that the proposed framework had excellent performance, achieving state-of-the-art performance on the proposed benchmark, ECGGenEval, including the signal-level, feature-level, and diagnostic-level evaluation. For example, the average Pearson correlation coefficients in the internal and external testing set are 0.7772 and 0.7287, outperforming the related approaches. Additionally, it is shown that the zero-padding strategy can play an important role in the proposed framework, beats the copy-padding strategy. In the future, it is necessary to study high-quality ECG and clinical validation, to let the proposed framework play an important role in clinical practice, which provides a novel feasible solution for long-term cardiac health monitoring.

## Supplementary information

Supplementary Information

## Data Availability

All datasets used in this study are openly available. PTB-XL: https://physionet.org/content/ptb-xl/1.0.3/, CPSC-2018: http://2018.icbeb.org/Challenge.html, CODE-test: https://zenodo.org/records/3765780.

## Code availability

The open-source code is publicly available at https://github.com/CHENJIAR3/MCMA.
