# Supplementary material for: Multi-channel masked autoencoder and comprehensive evaluations for reconstructing 12-lead ECG from arbitrary single-lead ECG
Source: NPJ Cardiovasc Health. 2024 Dec 4;1:34. doi: 10.1038/s44325-024-00036-4 (PMC12912321; doi:10.1038/s44325-024-00036-4)
Supplement: Supplementary file 1 — Supplementary Information [file 44325_2024_36_MOESM1_ESM.pdf]

Multi-Channel Masked Autoencoder and Comprehensive Evaluations for Reconstructing 12-Lead ECG from Arbitrary Single-Lead ECG

Jiarong Chen[1,2,3], Wanqing Wu[2], Tong Liu[4], Shenda Hong[1,5,6*]

[1*]National Institute of Health Data Science, Peking University, Beijing 100191, China.

[2]School of Biomedical Engineering, Sun Yat-sen University, Shenzhen 518107, Guangdong, China.

[3]Department of Micro/Nano Electronics and MoE Key Lab of Artificial Intelligence, Shanghai Jiao Tong University, Shanghai 200240, China.

[4]Tianjin Key Laboratory of Ionic-Molecular Function of Cardiovascular Disease, Department of Cardiology, Tianjin Institute of Cardiology, Second Hospital of Tianjin Medical University, Tianjin 300211, China.

[5*]Institute of Medical Technology, Health Science Center of Peking University, Beijing 100191, China .

[6*]Institute for Artificial Intelligence, Peking University, Beijing 100871, China.

*Corresponding author(s). E-mail(s): hongshenda@pku.edu.cn;
Contributing authors: chenjr356@gmail.com;
wuwanqing@mail.sysu.edu.cn; liutong@tmu.edu.cn;

This study do not focus on the hyperparameter searching, but they may have a bit of influence on the 12-lead ECG reconstruction, like kernel size and window size, as seen in Supplementary Table 1 and Supplementary Table 2. Since the proposed contributions could be proven with the value setting, the experimental results are provided as a reference for the related works.

Therefore, based on the above experimental results from Supplementary Table 1 and Supplementary Table 2, it is reasonable and acceptable for this study to take 5 and 2 as the kernel size and window size, respectively. The reconstruction performance with this setting could outperform other setting.

Supplementary Table 1: Supplementary Table 1: The reconstruction performance with various kernel size(k), and the used kernel size is 5

k	FLOPs	Params	PTB-XL		CPSC2018	
			MSE	PCC	MSE	PCC
1	172.4M	1.4120M	0.5745	0.0341	0.5151	0.0839
3	502.3M	4.2078M	0.7695	0.0175	0.7206	0.0658
5	832.1M	7.0036M	0.7772	0.0175	0.7287	0.0654
7	1162M	9.7994M	0.7698	0.0177	0.7157	0.0660
9	1492M	12.5952M	0.7719	0.0176	0.7162	0.0660
11	1822M	15.3910M	0.7724	0.0176	0.7224	0.0658
13	2151M	18.1868M	0.7698	0.0178	0.7237	0.0658
15	2481M	20.9826M	0.7740	0.0175	0.7218	0.0659
17	2811M	23.7784M	0.7676	0.0175	0.7135	0.0658
19	3141M	26.5743M	0.7684	0.0177	0.7152	0.0660

Supplementary Table 2: The reconstruction performance with various window size(s), and the used window size is 2

s	FLOPs	Params	PTB-XL		CPSC2018	
			MSE	PCC	MSE	PCC
1	14371M	7.0036M	0.7720	0.0174	0.7167	0.0655
2	832.1M	7.0036M	0.7772	0.0175	0.7287	0.0654
4	106.7M	7.0036M	0.7667	0.0177	0.7131	0.0660
